# Discontinuation of antidepressants after remission with antidepressant medication in major depressive disorder: a systematic review and meta-analysis

**DOI:** 10.1038/s41380-020-0843-0

**Published:** 2020-07-23

**Authors:** Masaki Kato, Hikaru Hori, Takeshi Inoue, Junichi Iga, Masaaki Iwata, Takahiko Inagaki, Kiyomi Shinohara, Hissei Imai, Atsunobu Murata, Kazuo Mishima, Aran Tajika

**Affiliations:** 1 Department of Neuropsychiatry, Kansai Medical University, Osaka, Japan; 2 Department of Psychiatry, University of Occupational and Environmental Health, Kitakyushu, Japan; 3 Department of Psychiatry, Tokyo Medical University, Tokyo, Japan; 4 Department of Neuropsychiatry, Molecules and Function, Ehime University Graduate School of Medicine, Shitsukawa, Toon, Ehime Japan; 5 Department of Neuropsychiatry, Faculty of Medicine, Tottori University, Yonago, Japan; 6 Adolescent Mental Health Service, Biwako Hospital, Otsu, Japan; 7 Department of Psychiatry, Shiga University of Medical Science, Otsu, Japan; 8 Department of Health Promotion and Human Behavior, Kyoto University Graduate School of Medicine and School of Public Health, Kyoto, Japan; 9 Department of Pathology of Mental Diseases, National Institute of Mental Health, National Center of Neurology and Psychiatry, Kodaira, Japan; 10 Department of Neuropsychiatry, Akita University Graduate School of Medicine, Akita, Japan; 11 Department of Psychiatry, Kyoto University Hospital, Kyoto, Japan

**Keywords:** Depression, Prognostic markers

## Abstract

A significant clinical issue encountered after a successful acute major depressive disorder (MDD) treatment is the relapse of depressive symptoms. Although continuing maintenance therapy with antidepressants is generally recommended, there is no established protocol on whether or not it is necessary to prescribe the antidepressant used to achieve remission. In this meta-analysis, the risk of relapse and treatment failure when either continuing with the same drug used to achieved remission or switching to a placebo was assessed in several clinically significant subgroups. The pooled odds ratio (OR) (±95% confidence intervals (CI)) was calculated using a random effects model. Across 40 studies (*n* = 8890), the relapse rate was significantly lower in the antidepressant group than the placebo group by about 20% (OR = 0.38, CI: 0.33–0.43, *p* < 0.00001; 20.9% vs 39.7%). The difference in the relapse rate between the antidepressant and placebo groups was greater for tricyclics (25.3%; OR = 0.30, CI: 0.17–0.50, *p* < 0.00001), SSRIs (21.8%; OR = 0.33, CI: 0.28–0.38, *p* < 0.00001), and other newer agents (16.0%; OR = 0.44, CI: 0.36–0.54, *p* < 0.00001) in that order, while the effect size of acceptability was greater for SSRIs than for other antidepressants. A flexible dose schedule (OR = 0.30, CI: 0.23–0.48, *p* < 0.00001) had a greater effect size than a fixed dose (OR = 0.41, CI: 0.36–0.48, *p* < 0.00001) in comparison to placebo. Even in studies assigned after continuous treatment for more than 6 months after remission, the continued use of antidepressants had a lower relapse rate than the use of a placebo (OR = 0.40, CI: 0.29–0.55, *p* < 0.00001; 20.2% vs 37.2%). The difference in relapse rate was similar from a maintenance period of 6 months (OR = 0.41, CI: 0.35–0.48, *p* < 0.00001; 19.6% vs 37.6%) to over 1 year (OR = 0.35, CI: 0.29–0.41, *p* < 0.00001; 19.9% vs 39.8%). The all-cause dropout of antidepressant and placebo groups was 43% and 58%, respectively, (OR = 0.47, CI: 0.40–0.55, *p* < 0.00001). The tolerability rate was ~4% for both groups. The rate of relapse (OR = 0.32, CI: 0.18–0.64, *p* = 0.0010, 41.0% vs 66.7%) and all-cause dropout among adolescents was higher than in adults. To prevent relapse and treatment failure, maintenance therapy, and careful attention for at least 6 months after remission is recommended. SSRIs are well-balanced agents, and flexible dose adjustments are more effective for relapse prevention.

## Introduction

Major depressive disorder (MDD) is among the most common psychiatric disorders. It is a chronic condition associated with significant functional impairment [1, 2]. Recently, treatment goals have focused on full recovery from depression, entailing both remission of depressive symptoms and restoration of vocational and interpersonal functions [3]. The relapse/recurrence of depressive symptoms after successful acute MDD treatment is common and is a significant clinical concern. The risk of relapse/recurrence is significantly reduced by continuation of antidepressant after acute treatment [4–6]. Several treatment guidelines recommend that patients with a major depressive episode continue antidepressant therapy for 4 to 9 months after successful acute phase treatment to prevent relapse/recurrence of the episode [7, 8] and up to 2 years or more of maintenance treatment at full therapeutic dose for patients with an increased risk of recurrence of MDD [9].

However, the meta-analysis used as the basis for these guidelines contains information about antidepressant polypharmacy, antidepressants plus psychotherapy, and data on classes of antidepressants used for maintenance treatment that are different than those used during acute phase treatment. Consequently, it is difficult for clinicians to meaningfully interpret those data. The most common treatment to prevent relapse/recurrence of MDD in the maintenance phase is to continue the same antidepressant medication that the subjects responded to during the acute treatment phase, so called “enrichment design” [10]. To date, only one meta-analysis has focused on such design. The meta-analysis conducted by Borges et al. in 2014 included 15 studies submitted to FDA [11]. However, they did not take potential risk factors of relapse into consideration. In general, recurrent episodes, duration of maintenance period after reaching remission, subject age, type of antidepressant, dosing schedule, and discontinuation method are considered to be risk factors of relapse [9]. Unfortunately, the relevance of these important clinical factors has not yet been fully elucidated. There are two other meta-analyses conducted recently [4, 12], but they included various interventions and pooled heterogeneous designs together without considerations to it. Combining the results of the different designs and treatments may not reflect the result of standard relapse prevention studies of antidepressants. On the other hand, of the 40 randomized controlled trials (RCTs) of enrichment design that we judged reasonable for inclusion in our meta-analysis, no more than 15 were included in either meta-analysis. Furthermore, their meta-analysis did not analyze acceptability and tolerability, which are important outcomes when evaluating usefulness of drug [9]. Therefore, this meta-analysis was performed to determine whether or not the antidepressant treatment therapy should be continued after remission, taking into account the influence of various clinically factors, focusing on studies that compared the relapse/recurrence rate of patients continuing the drug which they had achieved remission with vs a placebo.

## Methods

### Criteria for considering studies for this review

Double-blind RCTs were included. There are four types of RCT designs used to assess the effectiveness of long-term treatment [10]. Among them, we only included “discontinuation trial design” [13] (so called “enrichment design” [10]), in which patients who responded to an active drug in unblinded acute treatment phase were randomized to either continue taking the active drug or switch to placebo. All participants were diagnosed with MDD through the following operationalized criteria: Feighner criteria [14], Research Diagnostic Criteria [15], DSM-III, DSM-III-R, DSM-IV, DSM-5 [16], and ICD-10 [17]. We excluded the studies focused on bipolar disorder, personality disorder, substance use disorder, refractory depression, seasonal depression, perinatal depression, and other types of depression caused by certain physical diseases. Studies were required to have durations of at least 12 weeks after randomization. Types of interventions were presents in Supplementary Material.

### Search methods for identification of studies and management

An electronic search of Cochrane CENTRAL (until June 14, 2018), MEDLINE (until June 12, 2018), and EMBASE (until October 10, 2018) was carried out. Search terms can be found in supplemental data (Table S1). Two reviewers independently performed the literature search and reviewed all identified publications. Any disagreement was resolved by discussion with another reviewer.

Our outcomes were “relapse,” “all-cause dropout (acceptability),” and “dropout due to adverse events (tolerability).” The relapse rate at the respective endpoints of each study was used for this meta-analysis.

In the literature, relapse is defined as a return to case level symptoms during remission while recurrence is defined as a return to case level symptoms during recovery [18]. In this study, the term “relapse” is used for convenience rather than “recurrence”, as few studies have continued therapy for more than 6 months after remission before randomization. Data extraction and assessment methods were presented in Supplementary Material.

### Data analysis

We conducted pairwise meta-analysis in comparing all antidepressants vs placebo. A random effects model was used to synthesize the data. We obtained the odds ratio (OR) and risk difference (RD) for active treatments vs placebo from dichotomous data using Review Manager (RevMan) 5.3 [19]. When the random effects model showed significant differences between groups, the number needed to treat (NNT) was estimated.

We also assessed the effect of each factor on relapse, acceptability, or tolerability by meta-regression and/or subgroup analysis. The factors used in these analyses are presented in the Supplementary Material. Subgroup analysis was only performed for factors with categorical variables, and meta-regression analysis was performed only when the differences between groups were significant in the random effects model. Meta-regression was performed using Stata 16 (StataCorp).

Other details regarding data extraction, assessment, and analysis are also described in the Supplementary Material.

## Results

### Search and study characteristics

**Fig. 1:**
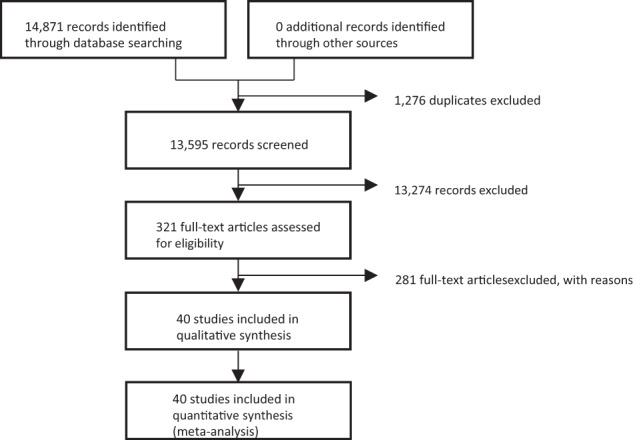
PRISMA flow diagram of the literature search.

The screening and selection process are summarized in a Prisma flow chart (Fig. 1). Searches of the MEDLINE, Cochrane Library, and Embase databases yielded 14,871 reports, respectively. Of the 13,595 remaining citations, we excluded 13,274 as not meeting study inclusion/exclusion criteria. The other 321 full reports were reviewed in detail. From these, 281 were excluded for having participants with diagnoses other than MDD, participants randomized to drugs other than antidepressants that remitted during the acute phase (nonenrichment design), or trials performed in designs other than double-blind RCT. The remaining 40 studies with 8890 participants [20–59] were included in the meta-analysis. Table 1 summarizes the characteristics of the studies included. Figure S1 addresses the risk of bias assessment. The number of participants per study ranged from 22 to 548 (median: 230.5) and the mean maximum study duration was 42 weeks (range: 14–100, SD 18.3). The mean age of the participants in the studies included was 43.1 years (range 11.5–76.8, SD 12.5) except for studies by Doogan et al. [21] and Montgomery et al. 1993b, in which no average age was stated. Three studies included only adolescents or children [49, 51, 52] and three studies included only older subjects [37, 39, 50]. With regard to the antidepressant discontinuation method, 8 trials used the abrupt discontinuation method, 16 trials used the tapering method, and the rest did not mention the discontinuation method. Of the 40 trials, 7 continued the same antidepressant medication (continuation therapy) for more than 6 months and 14 continued for more than 3 months after remission in acute phase before randomization (Table 1).

**Table 1:** Description of included studies.

	Maintenance phase											Acute Phase			
Study (year)	Total subjects/ Region	Intervention	n	Age		%Female	Trial length (wks)	Relapse definition	Dose(mg)		Discontinuation method	Acute phase length (wks)	Severity of acute phase (mean)	Scale	Continuation after remission (wks)
Stein et al. (1980)	55	Amitriptyline	(29)	42.3	(12.8)	65.0	26	NA	100–150	Flexible	abrupt	8	25.1	HAMD21	2
	North America	Placebo	(26)				26								
Doogan et al. (1992)	295	Sertraline	(185)	NA	NA	NA	44	CGI-S > = 4	50–200	Flexible	abrupt	8	NA	NA	0
	Europe	Placebo	(110)				44								
Montgomery et al. (1993a)	135	Paroxetine	(68)	48.3	(8.4)	77.6	16/52	(1) CGI-S > = 4, (2) deterioration of the CGI by 2 points or more, (3) patients meet DSM-III-R criteria for MDD of 2 weeks, (4) patients need antidepressant (5) depressive symptomatology was present for more than 7 days	20–30	Flexible	NA	8	26.9	HAMD21	0
	Europe	Placebo	(67)	45.9	(9.0)	79.4	52								
Montgomery et al. (1993b)	147	Citalopram	(105)	NA	NA	NA	24	MADRS score of 22 or more as a measure of the return of the symptoms of depression	20/40	Fixed	NA	6	NA	MADRS	0
	Unclear	Placebo	(42)				24								
Robert et al. (1995)	226	Citalopram	(152)	46.5	NA	68.9	24	MADRS > = 25 and the clinical judgment of the investigator	20/60	Fixed	NA	8	NA	MADRS	0
	Europe	Placebo	(74)	49.5		73.0	24								
Stewart et al. (1997)	32	Imipramine	(17)	38.0	(7.0)	66.0	26	2 consecutive weeks of a CGI -I > = 3 (compared with the pretreatment baseline).	150–400	Flexible	tapering	12	13.0	HAMD	26
	North America	Placebo	(15)				26								
Keller et al. (1998)	161	Sertraline	(77)	42.4	(9.7)	69.0	76	(1) DSM-III-R criteria for MDD for at least 3 weeks, (2) CGI-S > = 4, CGI-I > = 3, (4) an increase in HAM-D to a score of 4 or more than maintenance phase baseline.	50–200	Flexible	tapering	28	24.5	HAMD24	16
	Unclear	Placebo	(84)	40.8	(9.0)	62.0	76								
Reimherr et al. (1998)	395	Fluoxetine	(299)	40.5	(10.5)	80.2	14/38/50	Met the criteria for MDD (even if all symptoms were classified as mild) for at least 2 weeks at any assessment during the double-blind phase or HAM-D > = 14 for 3 consecutive weeks	20	Fixed	NA	12–14	about 20	HAMD	0
	North America	Placebo	(96)	40.2	(10.5)	65.9	50								
Terra et al. (1998)	204	Fluvoxamine	(110)	45.0	(11.4)	78.0	52	Definition of DSM-III-R or suicide attempt	100	Fixed	tapering	26	24.2	HAMD21	19
	Europe	Placebo	(94)	44.5	(10.7)	70.0	52								
Feiger et al. (1999)	131	Nefazodone	(65)	42.0	NA	71.0	36	Either HAMD17 > = 18 for two consecutive visits or lack of efficacy	100–600	Flexible	NA	16	24.2	HAMD	0
	North America	Placebo	(66)	40.0		72.0	36								
Versiani et al. (1999)	286	Reboxetine	(145)	42.3	(12.2)	67.4	46	HAMD > = 18 and 50%>=increase	4–8	Flexible	NA	6	29.6	HAMD21	0
	Cross-Continental	Placebo	(111)	43.4	(11.6)	79.3	46								
Dekker et al. (2000)	30	Fluoxetine	(15)	37.0	(10.0)	61.9	22	HAM-D > = 14	20	Fixed	NA	up to 16	NA	NA	0
	Europe	Placebo	(15)				22								
Rouillon et al. (2000)	214	Milnacipran	(104)	44.6	(10.0)	68.2	48	Major depressive episode according to DSM III-R criteria and a minimum score of 18 on HAM-D with the need to treat the recurrence	100	Fixed	NA	26	25.1	HAMD21	19
	Europe	Placebo	(110)	46.1	(10.2)	66.3	48								
Schmidt et al. (2000)	311	Fluoxetine	(189)	42.0	(11.2)	63.9	25	SCID-P MDD and CGI increase > =2	20	Fixed	abrupt	13	NA	NA	0
	North America	Placebo	(122)	41.7	(11.3)	70.9	25								
Dalery et al. (2001)	185	Tianeptine	(111)	42.2	NA	64.9	66	Either HAMD17 > = 15 (CGI > = 4) or clinical confirmation	37.5	Fixed	NA	6	23.3	HAMD17	0
	Europe	Placebo	(74)	44.1		65.7	66								
Gilaberte et al. (2001)	140	Fluoxetine	(70)	43.8	NA	78.6	52	Meeting DSMIIR criteria for MDD and having HAMD17 score > = 18 and CGI score > = 4	20	Fixed	NA	32	24.0	HAMD17	24
	Europe	Placebo	(70)	44.4		78.6	52								
Hochstrasser et al. (2001)	264	Citalopram	(132)	42.4	(11.5)	75.0	48	MADRS > = 22	20/60	Fixed	NA	25	30.6	MADRS	16
	Europe	Placebo	(132)	43.8	(9.7)	67.4	48								
Thase et al. (2001)	156	Mirtazapine	(76)	40.7	(11.3)	48.8	40	clinical judgment	30/45	Fixed	abrupt	12	22.7	HAMD17	2,4
	North America	Placebo	(80)	40.1	(12.0)	52.6	40								
Klysner et al. (2002)	121	Citalopram	(60)	75.0	NA	72.0	48	MADRS > = 22	20/40	Fixed	NA	24	27.0	MADRS	16
	Europe	Placebo	(61)	74.0	NA	82.0	48								
Weihs et al. (2002)	423	Bupropion	(210)	39.9	NA	64.0	44	Determined by the investigator to be necessary for the treatment of depression	300	Fixed	NA	8	NA	NA	0
	North America	Placebo	(213)	39.4	NA	66.0	44								
Wilson 2003 et al. (2003)	113	Sertraline	(56)	76.8	(7.0)	75.4	48/100	HAMD score of 13 or over as well as meeting DSM–III–R criteria for major depressive disorder as determined by a trained psychiatrist.	50–150	Flexible	NA	8	20.4	HAMD17	16–20
	Europe	Placebo	(57)	76.6	(6.6)	66.1	100								
Emslie et al. (2004)	40	Fluoxetine	(20)	11.7	(2.5)	55.0	32	CDRS-S score of >40 with a 2-week history of worsening of depressive symptoms or relapse in the opinion of the physician	10–60	Flexible	abrupt	19	57.1	CDRS-R	0
	North America	Placebo	(20)	13.5	(2.4)	45.0	32								
Montgomery et al. (2004)	235	Venlafaxine	(112)	43.5	(11.2)	67.0	52	CGI-S > = 4	100–200	Flexible	tapering	26	25.2	HAMD21	19
	Cross-Continental	Placebo	(123)	43.8	(11.0)	71.0	52								
Rapaport et al. (2004)	274	Escitalopram	(181)	41.8	(11.9)	62.4	36	MADRS > = 22	10/20	Fixed	NA	8	14.9	MADRS	0
	North America	Placebo	(93)	42.9	(11.6)	60.2	36								
Simon et al. (2004)	318	Venlafaxine	(161)	41.0	NA	62.0	26	Either a combination of the reappearance of MDD (DSM-IV criteria) and a CGI-S > = 4, two consecutive CGI-S > = 4, or a final CGI-S > = 4	75/150/225	Fixed	tapering	8	24.5	HAMD21	0
	North America	Placebo	(157)	43.0	NA	66.0	26								
Fava et al. (2006)	278	Duloxetine	(136)	44.8	(11.9)	77.5	26	CGI-S increase of > =2 points compared with the randomization visit and meeting the MINI criteria for MDD for two consecutive visits	60	Fixed	tapering	12	23.7	HAMD17	0
	Cross-Continental	Placebo	(142)	45.7	(12.7)	67.6	26								
McGrath et al. (2006)	262	Fluoxetine	(131)	38.2	(10.9)	55.3	26/52	Using CGI-I but definition is not clear	40/60	Fixed	NA	12	17.7	HAMD17	0
	North America	Placebo	(131)				52								
Gorwood et al. (2007)	305	Escitalopram	(152)	72.0	NA	79.0	24	MADRS > = 22 or lack of efficacy by investigator	10/20	Fixed	tapering	12	31.1	MADRS	0
	Europe	Placebo	(153)	73.0	NA	78.0	24								
Kocsis et al. (2007)	267	Venlafaxine	(132)	42.6	NA	67.0	26/52	HAMD > 12 and 2 consecutive visit, HAMD reduction <50% from acute phase	75–300	Flexible	tapering	36	22.5	NA	26
	North America	Placebo	(135)	42.0	NA	69.0	52								
Cheung et al. (2008)	22	Sertraline	(13)	16.3	NA	78.0	52	Clinical judgment	25–200	Fixed	tapering	36	20.7	HAMD17	24
	North America	Placebo	(9)	15.2	NA	77.0	52								
Dobson et al. (2008)	49	Paroxetine	(28)	38.9	(10.0)	78.2	52	HAMD > = 14 or Psychiatric status rating > = 5 for two successive weeks.	10–50	Flexible	tapering	16	20.9	HAMD17	0
	North America	Placebo	(21)				52								
Emslie et al. (2008)	102	Fluoxetine	(50)	11.5	(2.8)	36.3	24	Either a one-time CDRS-R score > = 40 with worsening of depressive symptoms for at least 2 weeks, or a clinician determination that there was significant clinical deterioration suggesting that full relapse would be likely without altering treatment	10–40	Fixed	abrupt	12	57.6	CDRS-R	0
	North America	Placebo	(52)				24								
Goodwin et al. (2009)	339	Agomelatine	(165)	43.1	(10.3)	76.4	24	HAMD17 > = 16, any withdrawal for lack of efficacy [clinical judgment based on HAMD and CGI], [suicide or suicide attempt]	25/50	Fixed	abrupt	8/10	27.0	HAMD17	0
	Cross-Continental	Placebo	(174)	43.4	(10.9)	72.1	24								
Perahia et al. (2009)	288	Duloxetine	(146)	48.0	(12.3)	74.6	52	(1) They had a CGI-S score≧4 and met DSM-IV criteria for MDD for at least 2 weeks; (2) they had 3 consecutive visits that met re-emergence criteria or 10 total re-emergence visits; or (3) they discontinued the study with a reason of “lack of efficacy”	60	Fixed	tapering	34	23.1	HAMD17	24
	Cross-Continental	Placebo	(142)	47.1	(12.8)	68.5	52								
Rickels et al. (2010)	375	Desvenlafaxine	(190)	42.8	(11.8)	68.0	24	HAM-D17 > = 16 or CGI-I > = 6 at any office visit during the DB treatment phase or as withdrawal from the study because of an unsatisfactory response to treatment as determined by the investigator	200/400	Fixed	tapering	12	24.2	HAMD17	0
	Cross-Continental	Placebo	(185)	42.7	(12.3)	67.0	24								
Segal et al. (2010)	58	Various	(28)	41.9	(11.6)	67.0	78	relapse of DSM-IV MDD episode		Flexible	tapering		19.4	HAMD17	28
	North America	Placebo	(30)	45.8	(11.4)	71.0	78								
Boulenger et al. (2012)	400	Vortioxetine	(206)	45.1	(12.1)	62.5	64	MADRS total score > = 22 or an insufficient therapeutic response	5/10	Fixed	abrupt	12	32.3	MADRS	0
	Cross-Continental	Placebo	(194)	44.8	(12.4)	63.7	64								
Goodwin et al. (2013)	367	Agomelatine	(187)	46.0	(10.1)	79.4	24/42	HAMD17 > = 16, any withdrawal for lack of efficacy [clinical judgment based on HAMD and CGI], [suicide or suicide attempt]	25	Fixed	NA	8	26.3	HAMD17	0
	Europe	Placebo	(180)	45.3	(10.5)	76.5	42								
Rosenthal et al. (2013)	548	Desvenlafaxine	(272)	45.3	(13.0)	71.7	26	HAMD17 total score >16, discontinuation for unsatisfactory response, hospitalization for depression, suicide attempt, or suicide (any 1 or more)	50	Fixed	tapering	20	24.2	HAMD17	12
	Cross-Continental	Placebo	(276)	46.6	(13.0)	71.0	26								
Shiovitz et al. (2014)	348	Levomilnacipran	(235)	44.7	(12.7)	54.5	24	(1) MADRS > = 22 at two consecutive visits; (2) increase > =2 in the CGI-I relative to the double-blind baseline score at two consecutive visits; (3) discontinuation from the score of 4 or greater	40/80/120	Fixed	tapering	12	30.7	MADRS	0
	North America	Placebo	(113)	42.6	(12.0)	59.7	24								

*CGI* Clinical Global Impression Scale (*I* improved; *S* severity), *DSM* APA Diagnostic & Statistical Manual, *Dx* diagnosis, *HAMD* Hamilton Depression Rating Scale (17-, 21-, or 24-item versions), *MADRS* Montgomergy-Åsberg Depression Rating Scale, *MDD* major depressive disorder, *NA* information not available, *wks* weeks, *yr* years.

### Relapse, acceptability, and tolerability at study endpoint

**Fig. 2:**
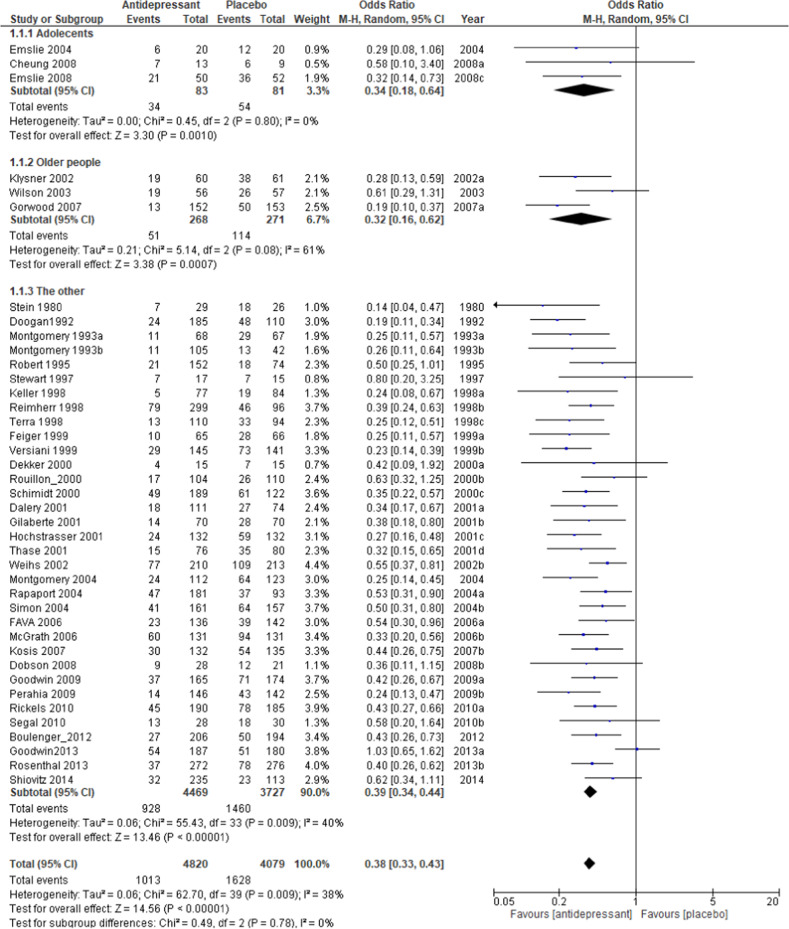
Meta-analysis of OR for study-defined relapse.

**Fig. 3:**
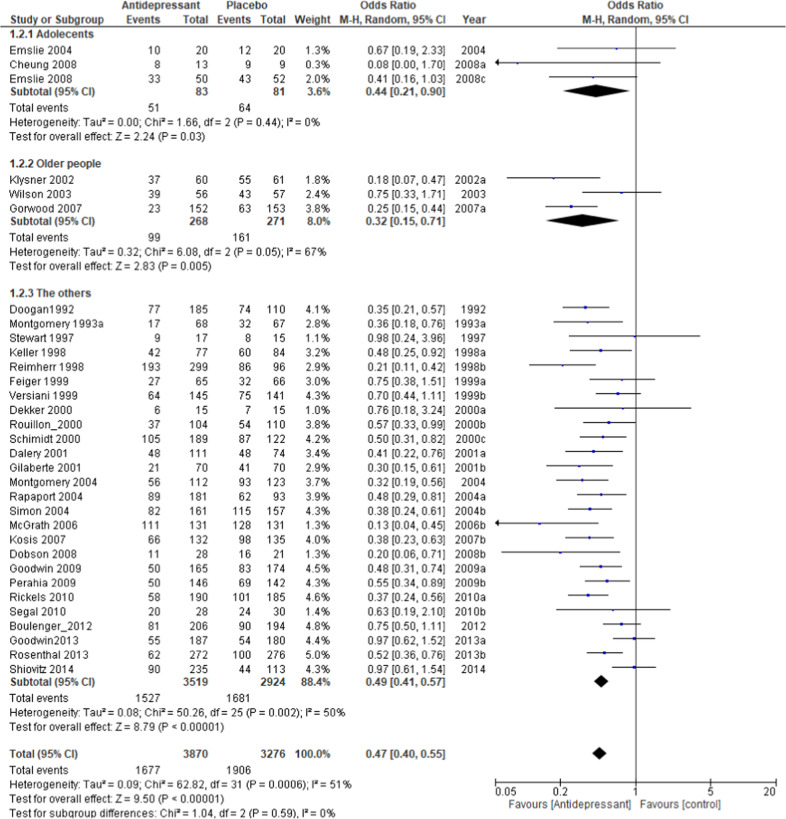
Meta-analysis of OR for acceptability.

**Fig. 4:**
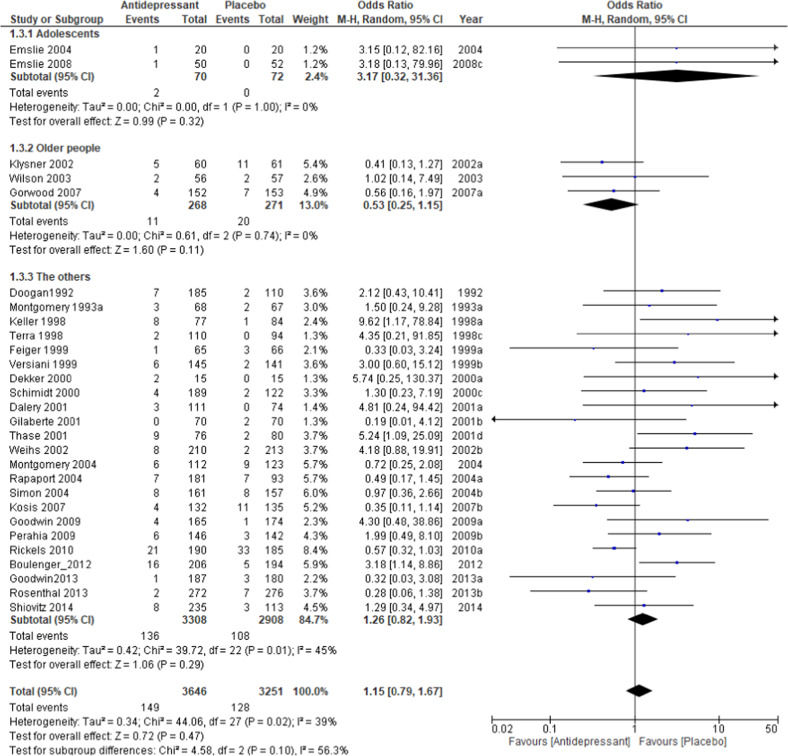
Meta-analysis of OR for tolerability.

The relapse rate at the respective endpoints of each study was used for this meta-analysis; the exception was Wilson et al. [39], whose endpoint at week 100 deviated significantly from the average of the other trials (40 weeks +15.9). For the Wilson et al. study, the relapse rate at 48 weeks was used for analysis. The pooled OR of relapse between the antidepressant and placebo groups performed with 40 studies and 8890 subjects was 0.38 (95% confidence intervals (CI), 0.33–0.43, *Z* = 14.56 *p* < 0.00001; Fig. 2), favoring antidepressant continuation over placebo. The RD of the relapse between antidepressant (20.9%) and placebo (39.7%) groups was 0.19 (95% CI, 0.16–0.22, *Z* = 14.01 p < 0.00001) and NNT was 6. In terms of acceptability, the pooled OR of 32 studies including 7146 subjects was 0.47 (95% CI, 0.40–0.55, *Z* = 9.50 *p* < 0.00001; Fig. 3), favoring antidepressant continuation over placebo. The RD of the rate of acceptability between antidepressant (43.3%) and placebo (58.2%) groups was 0.17 (95% CI, 0.14–0.20, *Z* = 10.68 *p* < 0.00001) and NNT was 7. For the rate of tolerability, pooled ORs of 28 studies with 6897 subjects was 1.15 (95% CI, 0.79–1.67, *Z* = 0.72 *p* = 0.47; Fig. 4) and RD was 0.01 (95% CI, −0.01 to 0.02, *Z* = 1.03 *p* = 0.30) without significant differences between antidepressant (4.1%) and placebo (3.9%) groups.

### Meta-regression and subgroup analyses

**Fig. 5:**
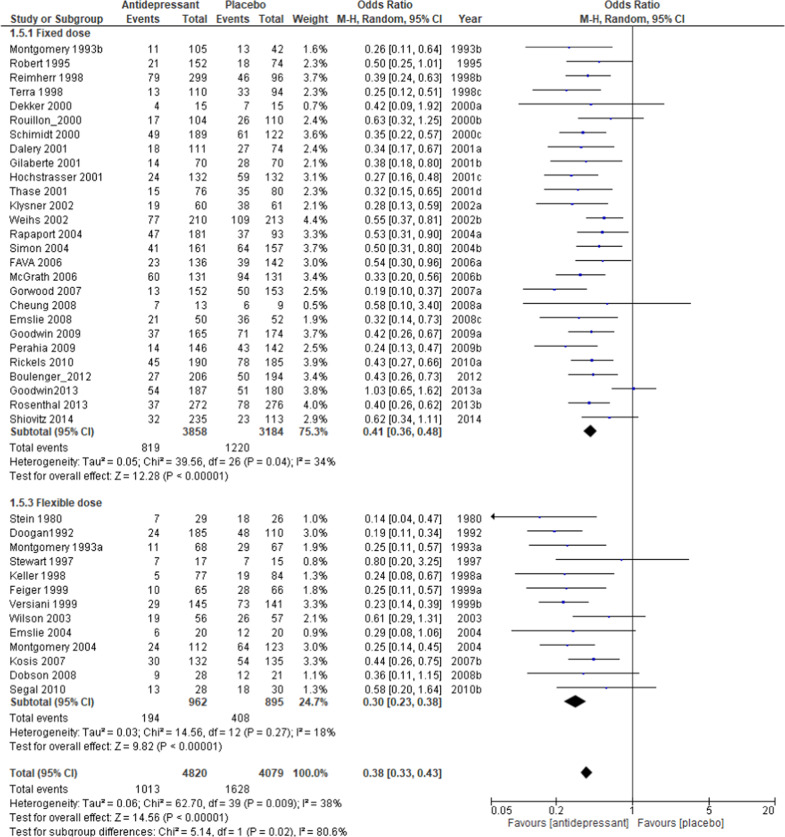
Meta-analysis of OR for relapse rate by dosing schedule.

Regarding the relapse rate, meta-regression analysis found the types of antidepressants (*p* = 0.04, *R*^2^ = 28.4%), dosing schedule (*p* = 0.03, *R*^2^ = 26.7%), and study year (beta = 0.03, *p* < 0.001, *R*^2^ = 46.5%) to be significantly associated with the outcome in meta-analysis. The difference in the relapse rate between the antidepressant and placebo groups was 25.3% (*N* = 4, *n* = 403, OR = 0.30, *p* < 0.00001) for classical antidepressants, 21.8% (*N* = 20, *n* = 3596, OR = 0.33, *p* < 0.00001) for SSRIs, and 16.0% (*N* = 15, *n* = 4842, OR = 0.44, *p* < 0.00001) for other newer agents (Fig. S2), 17.1% (*N* = 27, *n* = 7042, OR = 0.41, *p* < 0.00001) in the fixed dose setting and 25.5% (*N* = 13, *n* = 1857, OR = 0.30, *p* < 0.00001) for the flexible dose (Fig. 5). The relapse rate in adolescents was 66.7% for placebo and 41.0% for the antidepressant group, which was higher than the overall rate, with a large difference between the two groups (*N* = 3, *n* = 164, OR = 0.34, *p* = 0.0010; Fig. 2), although the meta-regression analysis showed no significant difference between age groups, probably due to the small number of trials. The relapse rate in older subjects was 42.1% for the placebo and 19.0% for the antidepressant groups, similar to overall results, but with a slightly larger difference (*N* = 3, *n* = 539, OR = 0.32, *p* = 0.0007; Fig. 2). Excluding the six trials specifically for the older people or adolescents, the results for the relapse rate (Fig. 2), acceptability rate (Fig. 3), and tolerability rate (Fig. 4) were comparable to the results for all trials. The difference of relapse rate between placebo and antidepressant was 4.6% higher in the abrupt discontinuation method (22.3%, *N* = 8, *n* = 1698, *OR* = 0.33, *p* < 0.00001) compared with the tapering method (17.7%, *N* = 15, *n* = 3488, OR = 0.38, *p* < 0.00001) without a significant difference in the meta-regression analysis. The relapse rate for recurrent depression only (*N* = 16, *n* = 3605, OR = 0.39, *p* < 0.00001) was comparable to the overall result. The difference of relapse rate by duration of continuous treatment after remission between placebo and antidepressant was 19.1% after 1 month (≤ 4w) of continuous treatment (mean 0.2w, median 0w, *N* = 26, *n* = 6231, OR = 0.38, *p* < 0.00001) and was equivalent to after more than 6 months (≥24w) of continuous treatment (17.5%, mean 27w, median 26w, *N* = 7, *n* = 869, OR = 0.40, *p* < 0.00001; Fig. S3). The difference of relapse rate between placebo and antidepressant group in studies with a duration of 1 year (more than 48w; mean 56w, median 52w) after randomization was 19.9% (*N* = 17, *n* = 3118, OR = 0.35, *p* < 0.00001) and for the 6-months period (22–26w; mean 25w, median 25w) the difference was 18.0% (*N* = 16, *n* = 3943, OR = 0.41, *p* < 0.00001).

The meta-regression analysis of the acceptability rate found only the type of antidepressant to be significantly associated with the outcome (*p* = 0.02, *R*^2^ = 29.7%). The difference in the relapse rate between the antidepressant and placebo groups was 13.5% (*N* = 3, *n* = 348, OR = 0.58, *p* < = 0.03) for classical antidepressants, 18.9% (*N* = 16, *n* = 2755, OR = 0.36, *p* < 0.00001) for SSRIs and 14.1% (*N* = 12, *n* = 3985, OR = 0.55, *p* < 0.00001) for other newer agents (Fig. S4). The rate of acceptability in adolescents was 79.0% for placebo and 61.4% for antidepressant (*N* = 3, *n* = 164, OR = 0.44, *p* = 0.03; Fig. 3), which was higher than the rates for older people, where the rate for placebo was 59.4% and antidepressants was 36.9% (*N* = 3, *n* = 539, OR = 0.32, *p* = 0.005; Fig. 3), and other people. The pooled OR of acceptability in each antidepressant discontinuation method was similar for the tapering (*N* = 12, *n* = 2688, OR = 0.45, *p* < 0.00001) and abrupt (*N* = 6, *n* = 1487, OR = 0.51, *p* < 0.00001) methods.

A subgroup analysis of the tolerability rate showed that the difference between antidepressants and placebo was numerically small, with 0.6 and 1.7% in the 1-year and 6-month trials, respectively (Fig. S5). There were no significant differences in tolerability by antidepressant type or age group (Fig. 3).

The other factors did not significantly affect the results in the meta-regression analysis.

### Publication bias

Figure S6 presents the funnel plots of relapse and tolerability with significant results from the meta-analysis. No publication bias was presented in the present study by Egger’s analysis [60].

## Discussion

This is the largest meta-analysis to date focusing on studies that address the frequently asked clinical question “ whether to continue the same antidepressant used to achieve remission or to discontinue” in remitted patients with a major depressive disorder. It was found that overall and, in most subgroups, the relapse rate was significantly lower (by about 20%) in the antidepressant group and NNT was around 6. It was also determined that 80% do not relapse when antidepressants are continued, although this decreases to 60% when discontinued; this can be interpreted as a 40% relapse rate. The rate of acceptability was 43% in the antidepressant group and 58% in the placebo group, with a 15% difference, both of which were 20% greater than the relapse rate. The rates of tolerability for the antidepressant continuation and the placebo group were both ~4%.

Although there were fewer RCTs on tricyclic antidepressants, the effect size of the relapse rates was greater for tricyclics, SSRIs, and other newer agents in that order compared with the placebo. Since the study year was inversely correlated with the effect size of the relapse rate, various factors associated with the study year may have influenced the results, including the type of antidepressant, while the effect size of acceptability was greater for SSRIs than for other antidepressants. Thus, SSRIs may be well-balanced for relapse prevention. Given that a flexible dose had a greater effect size for the relapse than fixed dose, symptom-based dose adjustment is recommended for relapse prevention. Both relapse and acceptability rates in adolescents were higher than in adults, and discontinuation of antidepressants was associated with a 26% higher relapse rate and an 18% higher acceptability rate than when continued. Relapse rates in older people were no different from adults but the effect size of the acceptability rate was greater. The relapse rate after 6-month and 1-year timeframes is similar in the subjects who continued the antidepressant medication while those who discontinued the medication showed a 2% increase in relapse rate after 1 year compared with after 6 months. This suggests that the relapse is more likely to occur by 6 months after discontinuation. Even in studies assigned after continuous treatment for more than 6 months after remission, antidepressants continuation has a lower relapse rate than placebo. The relapse rate difference between antidepressants and placebo for the 6-month was only 2% less than in studies with continuous treatment for less than 1 month. In a previous study by Reimherr et al. [27] there was no difference in the relapse rate between antidepressant discontinuation following 38 weeks of continuation therapy after remission and antidepressant continuation at 50 weeks, which suggests a 38-week maintenance therapy period to prevent relapse. Out of all the studies included in this meta-analysis, only the study by Reimherr et al. evaluated relapse rate after 38 weeks or more of continuous therapy, so we could not confirm it in this meta-analysis. However, our result suggests that maintenance therapy should be continued for at least 6 months after remission of the depressed episode. In addition, after half a year of continuous antidepressant treatment, maintaining antidepressants for another year showed a lower relapse rate than discontinuing them. In another study by Baldessarini et al. a strong correlation of shorter (<8 weeks) initial treatment and greater relapse risk compared with longer (≥12 weeks) initial treatment [61]. In our study, switching to placebo with an initial treatment less than 1 month after remission increases the relapse rate by 4% compared with switching after continuation of antidepressants for more than 6 months. However, the difference between placebo and the antidepressant continuation was similar in the short and long initial treatment periods. An analysis was performed for each discontinuation method while taking the effects of withdrawal symptoms into account. Even with these considerations, the rate of acceptability was still similar for both abrupt and tapering discontinuation methods. The tolerability rate is almost the same for both antidepressant and placebo groups in 1-year studies and 6-month studies, which may suggest that the dropout rate after 6-month is unlikely to increase.

Several previous meta-analyses assessed the risk of relapse risk during the maintenance period between continuation and discontinuation of antidepressants [4, 11, 12, 62–64]. Borges et al. focused on RCTs of enrichment design only [11], using 15 unpublished drug application studies submitted to the FDA. As a result, the relapse rate in the antidepressant arm was about 20% lower than that in the placebo arm and the difference in relapse rate between them after 6 months was maintained. Although we did not include the 15 studies of Borges’s study in our meta-analysis, their result was consistent with ours. As there was no information on whether the data of 15 studies were published in whole or in part, we decided not to include it to avoid the risk of duplication. Our results of the relapse rate were also similar to the meta-analysis by Sim et al. [12] However, their results showed a greater difference between placebo and antidepressants, as compared with our results, because of the differences in the RCTs included in the meta-analyses. Sim et al. included various types of maintenance therapy such as antidepressant monotherapy, polypharmacy, combination therapy (e.g., antidepressants plus psychotherapy, electroconvulsive therapy, and lithium) regardless of whether a study continued the same antidepressant used for acute treatment in the maintenance phase or switched to a new one. Moreover, they used the data from Borges et al.’s study which risks the possibility of duplicate analysis. In fact, a meta-analysis by Sim et al. showed that 6 of the 15 studies submitted to the FDA by Borges et al. had the same number of subjects as the other published studies included in their meta-analysis. In our study, we only included enrichment design studies that we identified to prevent duplication. In addition, we used the Cochrane CENTRAL and Embase database, which were not used in Sim’s and Glue’s study, to search for articles. As a result, we were able to find and include 3263 subjects (15 studies) [27, 33, 42, 46, 48–56, 58, 59] and 3886 subjects (18 studies) [20, 22, 33, 41–49, 51, 52, 55, 57–59] that were not included in the analysis by Sim et al. and Glue et al., respectively. This decision made our results more inclusive and rigorous in terms of assessing the efficacy of continuing the same antidepressant that patients respond to in the acute phase in the maintenance phase. Our results about adolescents and older people were similar to the results of previous meta-analyses that focused only on older subjects [63] or only on children/adolescents [62]. However, in our analysis, we were able to compare different age groups and get an overview from a more holistic perspective.

The results of this study have to be interpreted in the context of several limitations. First, factors such as the number of episodes, severity of episodes, chronic episodes, difficult-to-treat episodes, comorbid psychiatric conditions, pre-existing medical conditions, and residual symptoms are all thought to contribute to risk of relapse [9], but only few RCTs have evaluated them so far. Therefore, our meta-analysis could not take these factors into account. In our meta-analysis, the relapse rate for recurrent depression alone, in both the antidepressant and the placebo group, was comparable to the overall result. This suggests that episode frequency may play a more important role in assessing relapse compared with whether the episode was the first or recurrent. Second, enrichment designs were required to respond favorably to treatment response in acute phase and this could put a favorable bias on the antidepressant group. However, this result is considered to be relevant to actual clinical practice, as continuing the same drug used to achieve remission or discontinuing it are the two options that are commonly encouraged for maintenance therapy in actual clinical practice. Third, pharmacologic withdrawal of antidepressants could not be evaluated in our meta-analysis because relapse and dropout rates in the short 4–8 weeks could not be assessed. However, we believe that this risk is not significant because our subanalysis showed that dropout rates did not change in abrupt discontinuation trials and that withdrawal symptoms did not affect relapse in the short term results as seen in Borges et al. [11] Another important limitation is the inconsistent definition of relapse. The definitions vary in each study, making subgroup analysis difficult. However, it is believed that all studies were generally able to assess clinical recurrence/relapse.

In conclusion, relapse rates can be reduced to 20% through the continuation of the same antidepressant medication used to achieve remission, compared with 40% with antidepressant discontinuation. SSRIs are well-balanced agents, and flexible dose adjustments are more effective for relapse prevention. The relapse rate remained unchanged from 6 months to over 1 year in both the antidepressant and placebo groups. Neither group had an increase in relapse rate after 6 months, so more attention may be needed on the relapse rate in the first 6 months rather than 6 months after remission. All-cause dropout rates can also be reduced by 15% with continued use of antidepressants. This is unlikely to be affected by withdrawal symptoms of antidepressants. The tolerability is equally low with or without antidepressants and prolonged use of antidepressants does not seem to be related to withdrawal of treatment for side effects. Increased rates for relapse and/or dropout in adolescents and older subjects after discontinuing antidepressants may indicate that more attention should be given to these age groups. Maintenance therapy for at least 6 months after remission is recommended to prevent relapse, and attention should be given to relapses and treatment failure during this 6-month period.

Supplementary information is available at MP’s website.

## Supplementary information

Supplementary Figure and Table Legends

Supplemental Table 1

Supplemental Methods

Supplemental Figure 1

Supplemental Figure 2

Supplemental Figure 3

Supplemental Figure 4

Supplemental Figure 5

Supplemental Figure 6a

Supplemental Figure 6b
