# Supplementary material for: Discontinuation of antidepressants after remission with antidepressant medication in major depressive disorder: a systematic review and meta-analysis
Source: Mol Psychiatry. 2020 Jul 23;26(1):118–33. doi: 10.1038/s41380-020-0843-0 (PMC7815511; doi:10.1038/s41380-020-0843-0)
Supplement: Supplementary file 1 — Supplementary Figure and Table Legends [file 41380_2020_843_MOESM1_ESM.docx]

Supplemental Figure1

Title; Risk of bias assessment summary.

Legend; Review authors’ judgements about each risk of bias item for each included study.

Supplemental Figure2

Title; Meta-analysis of OR for relapse rate by types of antidepressants.

(No legend)

Supplemental Figure3

Title; Meta-analysis of OR for relapse rate by length of continuation treatment after remission.

(No legend)

Supplemental Figure4

Title; Meta-analysis of OR for acceptability rate by types of antidepressants

(No legend)

Supplemental Figure5

Title; Meta-analysis of OR for tolerability by trial length.

(No legend)

Supplemental Figure6-a

Title; Funnel plot of OR for study-defined relapse.

Supplemental Figure6-b

Title; Funnel plot of OR for acceptability

(No legend)

Supplemental table 1

Title; Database Search Terms

(No legend)
