# Supplementary material for: Discontinuation of antidepressants after remission with antidepressant medication in major depressive disorder: a systematic review and meta-analysis
Source: Mol Psychiatry. 2020 Jul 23;26(1):118–33. doi: 10.1038/s41380-020-0843-0 (PMC7815511; doi:10.1038/s41380-020-0843-0)
Supplement: Supplementary file 2 — Supplemental Table 1 [file 41380_2020_843_MOESM2_ESM.docx]

| **Ovid MEDLINE** | |  |
| --- | --- | --- |
| Search date (June 12, 2018) | |  |
| 1 | depression/ | 102,952 |
| 2 | mood disorders/ or depressive disorder/ or depression, postpartum/ or depressive disorder, major/ or dysthymic disorder/ or seasonal affective disorder/ | 110,831 |
| 3 | adjustment disorders/ | 4,112 |
| 4 | or/1-3 | 205,559 |
| 5 | recurrence/ | 170,280 |
| 6 | (recur$ or relaps$ or recrudesc$).ti,ab. | 577,178 |
| 7 | (maintenance$ or prophyla$ or prevent$ or continu$ or discontinu$).ti,ab | 2,149,803 |
| 8 | or/5-7 | 2,674,158 |
| 9 | randomized controlled trials as topic/ | 116,974 |
| 10 | randomized controlled trial.pt. | 463,264 |
| 11 | controlled clinical trial.pt. | 92,458 |
| 12 | randomi#ed.ab. | 435,168 |
| 13 | placebo$.ab. | 174,143 |
| 14 | randomly.ab. | 251,175 |
| 15 | trial.ti. | 160,153 |
| 16 | or/9-15 | 1,000,085 |
| 17 | (animals not (humans and animals)).sh. | 4,437,393 |
| 18 | 16 not 17 | 908,130 |
| 19 | 4 and 8 and 18 | 5,718 |

| **CENTRAL** | |  |
| --- | --- | --- |
| Search date (June 14, 2018) | |  |
| 1 | depress* | 76,356 |
| 2 | dysthymi* | 736 |
| 3 | "adjustment disorder*" | 368 |
| 4 | "mood disorder*" | 2,286 |
| 5 | "affective disorder*" | 2,478 |
| 6 | "affective symptoms" | 649 |
| 7 | depressi*.ti,ab. | 0 |
| 8 | (affective near/2 dis*) | 2,655 |
| 9 | (mood near/2 dis*) | 3,028 |
| 10 | (depress$ near/3 (patient* or symptom* or disorder*)) | 4 |
| 11 | #1 or #2 or #3 or #4 or #5 or #6 or #7 or #8 or #9 or #10 | 78,346 |
| 12 | recur* | 49,730 |
| 13 | relaps* | 27,984 |
| 14 | recrudesc* | 416 |
| 15 | maintenance | 30,111 |
| 16 | prophyla* | 27,560 |
| 17 | continuation | 3,370 |
| 18 | #8 or #9 or #10 or #11 or #12 or #13 | 120,297 |
| 19 | prevent* near recur* or relaps* or remis* or episode* | 70,914 |
| 20 | #7 and #14 and #15 | 3,542 |

| **EMBASE** | |  |
| --- | --- | --- |
| Search date (October 10, 2018) | |  |
| 1 | 'depression'/exp OR 'agitated depression'/exp OR 'atypical depression'/exp OR 'depressive psychosis'/exp OR 'dysthymia'/exp OR 'endogenous depression'/exp OR 'involutional depression'/exp OR 'major depression'/exp OR 'masked depression'/exp OR 'melancholia'/exp OR 'mixed anxiety and depression'/exp OR 'mixed depression and dementia'/exp OR 'mourning syndrome'/exp OR 'organic depression'/exp OR 'postoperative depression'/exp OR 'premenstrual dysphoric disorder'/exp OR 'pseudodementia'/exp OR 'puerperal depression'/exp OR 'reactive depression'/exp OR 'recurrent brief depression'/exp OR 'seasonal affective disorder'/exp | 439,052 |
| 2 | 'mood disorder'/exp | 477,486 |
| 3 | 'adjustment disorder'/exp | 3,964 |
| 4 | #1 OR #2 OR #3 | 635,154 |
| 5 | 'recurrent disease'/exp OR 'relapse'/exp | 283,327 |
| 6 | 'recur$' OR 'relaps$' OR 're- crudesc$':ti,ab | 245,479 |
| 7 | 'maintenance$' OR 'pro- phyla$' OR 'continuation':ti,ab | 385,280 |
| 8 | (prevent$ NEAR/7 recur$) OR 're- laps$' OR 'remis$' OR 'episode$':ti,ab | 279,395 |
| 9 | #5 OR #6 OR #7 OR #8 | 1,024,295 |
| 10 | 'phase 3 clinical trial'/exp OR 'phase 4 clinical trial'/exp | 38,446 |
| 11 | 'double blind procedure'/exp | 153,360 |
| 12 | 'single blind procedure'/exp | 32,596 |
| 13 | 'triple blind procedure'/exp | 191 |
| 14 | 'randomization'/exp | 79,496 |
| 15 | 'controlled study'/exp | 6,342,993 |
| 16 | 'placebo'/exp | 329,851 |
| 17 | 'placebo$':ti,ab | 277,252 |
| 18 | 'random$':ti,ab | 277,203 |
| 19 | 'randomi?ed controlled trial$':ti,ab | 184,845 |
| 20 | (clinical NEAR/3 trial$):ti,ab | 456,342 |
| 21 | (('singl$' OR 'doubl$' OR 'trebl$' OR 'tripl$') NEAR/3 ('blind$' OR 'mask$')):ti,ab | 194,199 |
| 22 | 'randomized controlled trial'/exp | 516,062 |
| 23 | #10 OR #11 OR #12 OR #13 OR #14 OR #15 OR #16 OR #17 OR #18 OR #19 OR #20 OR #21 OR #22 | 7,126,166 |
| 24 | (animal:de OR nonhuman:de) NOT (human:de AND (animal:de OR nonhuman:de)) | 5,913,479 |
| 25 | #23 NOT #24 | 4,941,549 |
| 26 | #4 AND #9 AND #25 | 19,397 |
| 27 | #26 AND [embase]/lim | 18,787 |
| 28 | #27 NOT ([embase]/lim AND [medline]/lim) | 5,611 |

Supp table 1; Database Search Terms
