# Supplementary material for: Discontinuation of antidepressants after remission with antidepressant medication in major depressive disorder: a systematic review and meta-analysis
Source: Mol Psychiatry. 2020 Jul 23;26(1):118–33. doi: 10.1038/s41380-020-0843-0 (PMC7815511; doi:10.1038/s41380-020-0843-0)
Supplement: Supplementary file 3 — Supplemental Methods [file 41380_2020_843_MOESM3_ESM.docx]

**Supplementary material**

Types of interventions

The following 31 antidepressants were included: Agomelatine, Amitriptyline, Amoxapine, Bupropion, Citalopram, Clomipramine, Desvenlafaxine, Doslepin, Duloxetine, Escitalopram, Fluoxetine, Fluvoxamine, Imipramine, Levomilnacipran, Lofepramine, Maprotiline, Mianserin, Milnacipran, Mirtazapine, Nefazodone, Nortriptyline, Paroxetine, Reboxetine, Sertraline, Setiptiline, Tianeptine, Trazodone, Trimipramine, Venlafaxine, Vilazodone, Vortioxetine. The selection of these antidepressants was based on a previous meta-analysis^18^ and other antidepressants commonly prescribed worldwide. Only studies looking at antidepressant monotherapy were included. Studies of combination antidepressant and electroconvulsive therapy or psychotherapy were excluded.

Data extraction

Two reviewers independently extracted the data. Any disagreement was resolved by discussion with another reviewer.

Risk of bias assessment

We assessed the risk of bias of each study using five domains of the Cochrane Risk of Bias tool namely, random sequence generation, allocation concealment, blinding of participants and personnel, blinding of outcome assessment, and incomplete outcome data. A table was generated using RevMan 5.3.

Assessment of reporting biases

To control for potential publication bias, the funnel plots were presented and formally analyzed by the method of Egger et al.,^19^ which is based on a linear regression of standard normal deviation of the OR on the inverse of the standard error of the OR.

Assessment of heterogeneity

We assessed statistical heterogeneity using I^2^ statistic.

Factors used in the meta-regression and/or subgroup analysis

(a) Age group: Children and adolescents, older people, or others; (b) Sex: Percentage of female subjects; (c) Types of antidepressants: Classical antidepressants, SSRIs, or other newer agents; (d) Discontinuation methods: Abrupt discontinuation or tapering; (e) Dosing schedule: Flexible or fixed; (f) Length of continuous treatment before randomization; (g) Total length of acute period and continuous treatment before randomization; (h) Length of maintenance treatment after randomization; (i) Research area: United States, Europe, or other; (j) Study year; and (k) Limitation to recurrent depression.
