# Supplementary material for: Discontinuation of antidepressants after remission with antidepressant medication in major depressive disorder: a systematic review and meta-analysis
Source: Mol Psychiatry. 2020 Jul 23;26(1):118–33. doi: 10.1038/s41380-020-0843-0 (PMC7815511; doi:10.1038/s41380-020-0843-0)
Supplement: Supplementary file 4 — Supplemental Figure 1 [file 41380_2020_843_MOESM4_ESM.pdf]

	Random sequence generation (selection bias)	Allocation concealment (selection bias)	Blinding of participants and personnel (performance bias)	Blinding of outcome assessment (detection bias)	Incomplete outcome data (attrition bias)
Boulenger_2012	+	+	+	+	?
Cheung 2008	+	?	+	+	−
Dalery 2001	?	?	+	+	+
Dekker 2000	?	+	+	+	+
Dobson 2008	?	?	+	+	−
Doogan1992	?	?	+	+	+
Emslie 2004	+	?	+	+	+
Emslie 2008	+	?	+	+	+
FAVA 2006	?	?	+	+	−
Feiger 1999	?	?	+	+	+
Gilaberte 2001	?	?	+	+	−
Goodwin 2009	?	?	+	+	−
Goodwin2013	?	?	+	+	−
Gorwood 2007	?	?	+	+	−
Hochstrasser 2001	?	?	+	+	+
Keller 1998	?	?	+	+	+
Klysner 2002	?	?	+	+	+
Kosis 2007	?	?	+	+	+
McGrath 2006	+	?	+	+	+
Montgomery 1993a	?	?	+	+	+
Montgomery 1993b	?	?	+	+	+
Montgomery 2004	?	?	+	+	+
Perahia 2009	?	?	+	+	+
Rapaport 2004	?	?	+	+	+
Reimherr 1998	?	?	+	+	+
Rickels 2010	?	?	+	+	+
Robert 1995	?	?	+	+	?
Rosenthal 2013	?	?	+	+	+
Rouillon_2000	?	?	+	?	−
Schimidt 2000	?	?	+	+	+
Segal 2010	+	?	+	+	+
Shiovitz 2014	+	+	+	+	+
Simon 2004	?	?	+	+	−
Stein 1980	?	?	+	+	?
Stewart 1997	?	?	+	+	+
Terra 1998	?	?	+	+	+
Thase 2001	?	?	+	+	+
Versiani 1999	?	?	+	+	+
Weihs 2002	?	?	+	+	?
Wilson 2003	+	+	+	+	−
