# Supplementary figures and images for: Discontinuation of antidepressants after remission with antidepressant medication in major depressive disorder: a systematic review and meta-analysis
Source: Mol Psychiatry. 2020 Jul 23;26(1):118–33. doi: 10.1038/s41380-020-0843-0 (PMC7815511; doi:10.1038/s41380-020-0843-0)

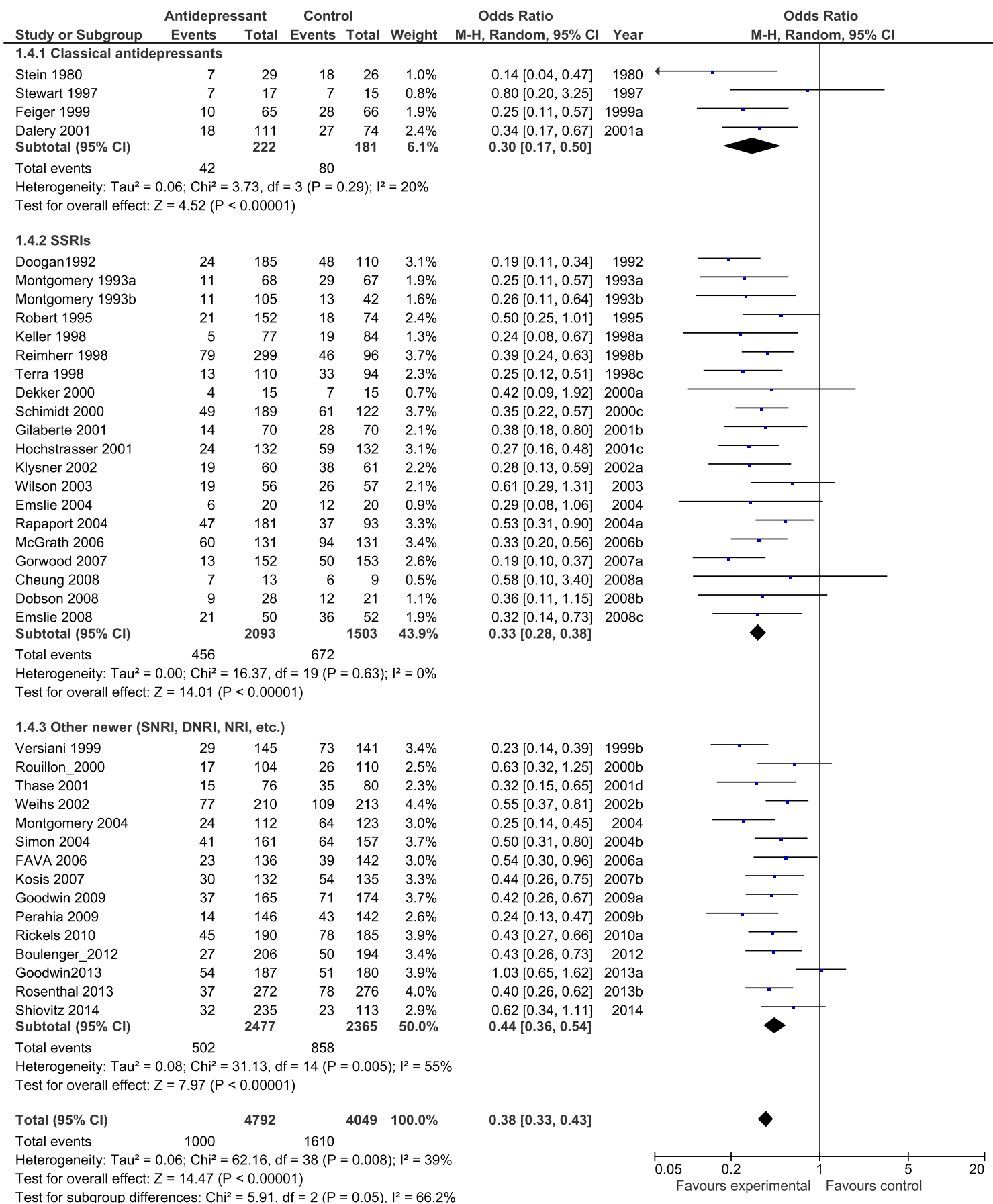

Supplement: Supplementary file 5 — Supplemental Figure 2 [file 41380_2020_843_MOESM5_ESM.pdf]

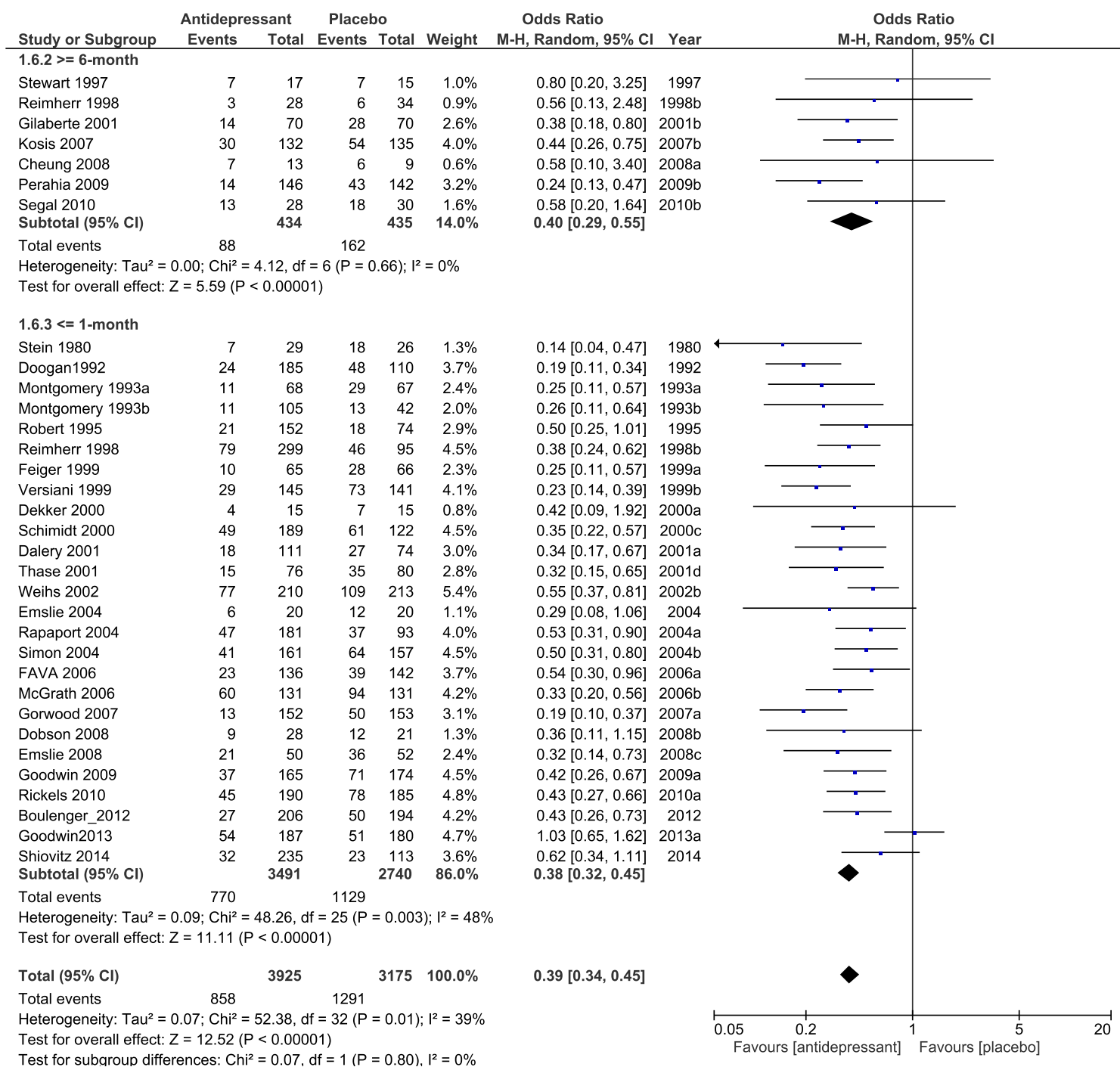

Supplement: Supplementary file 6 — Supplemental Figure 3 [file 41380_2020_843_MOESM6_ESM.pdf]

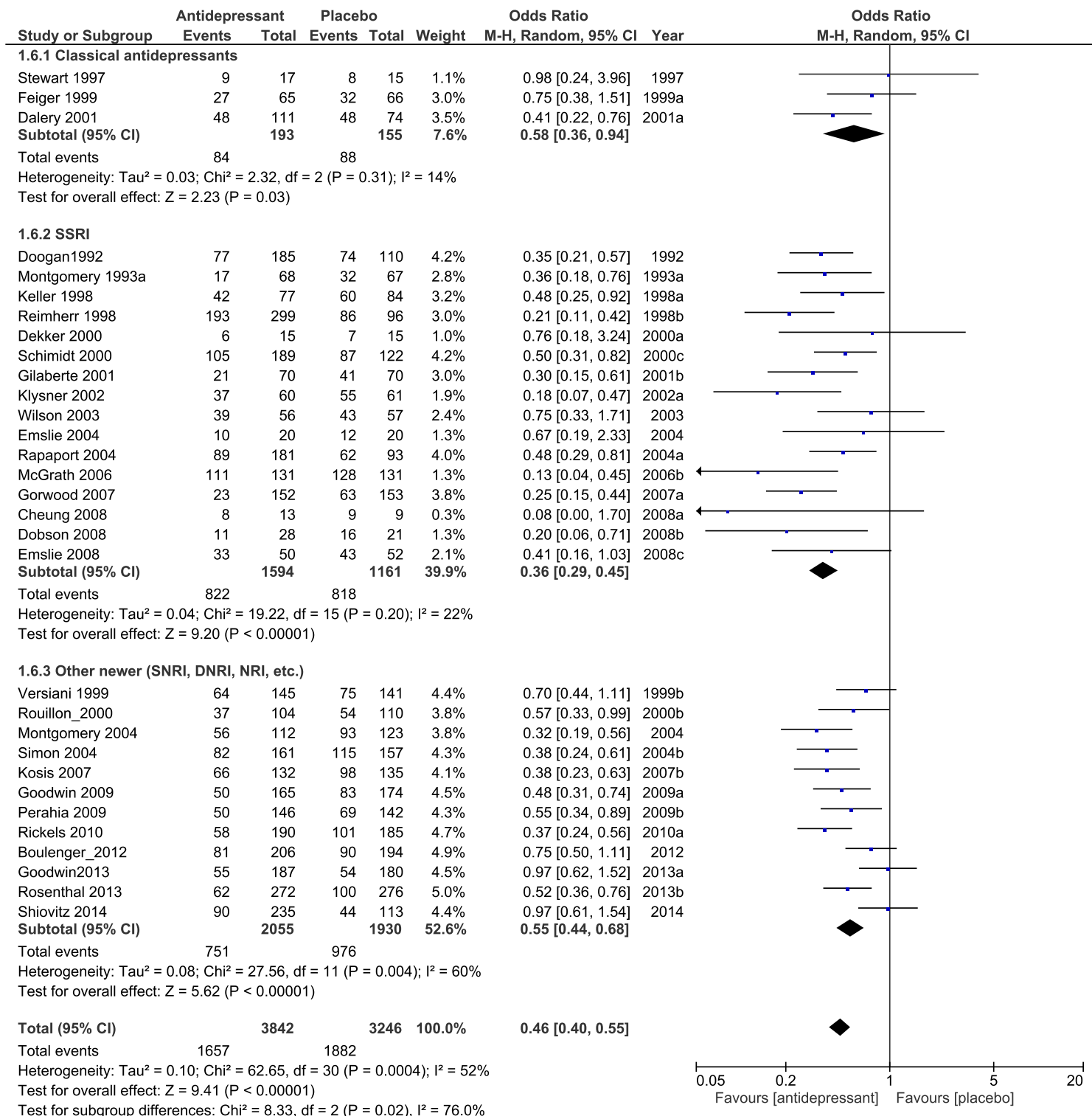

Supplement: Supplementary file 7 — Supplemental Figure 4 [file 41380_2020_843_MOESM7_ESM.pdf]

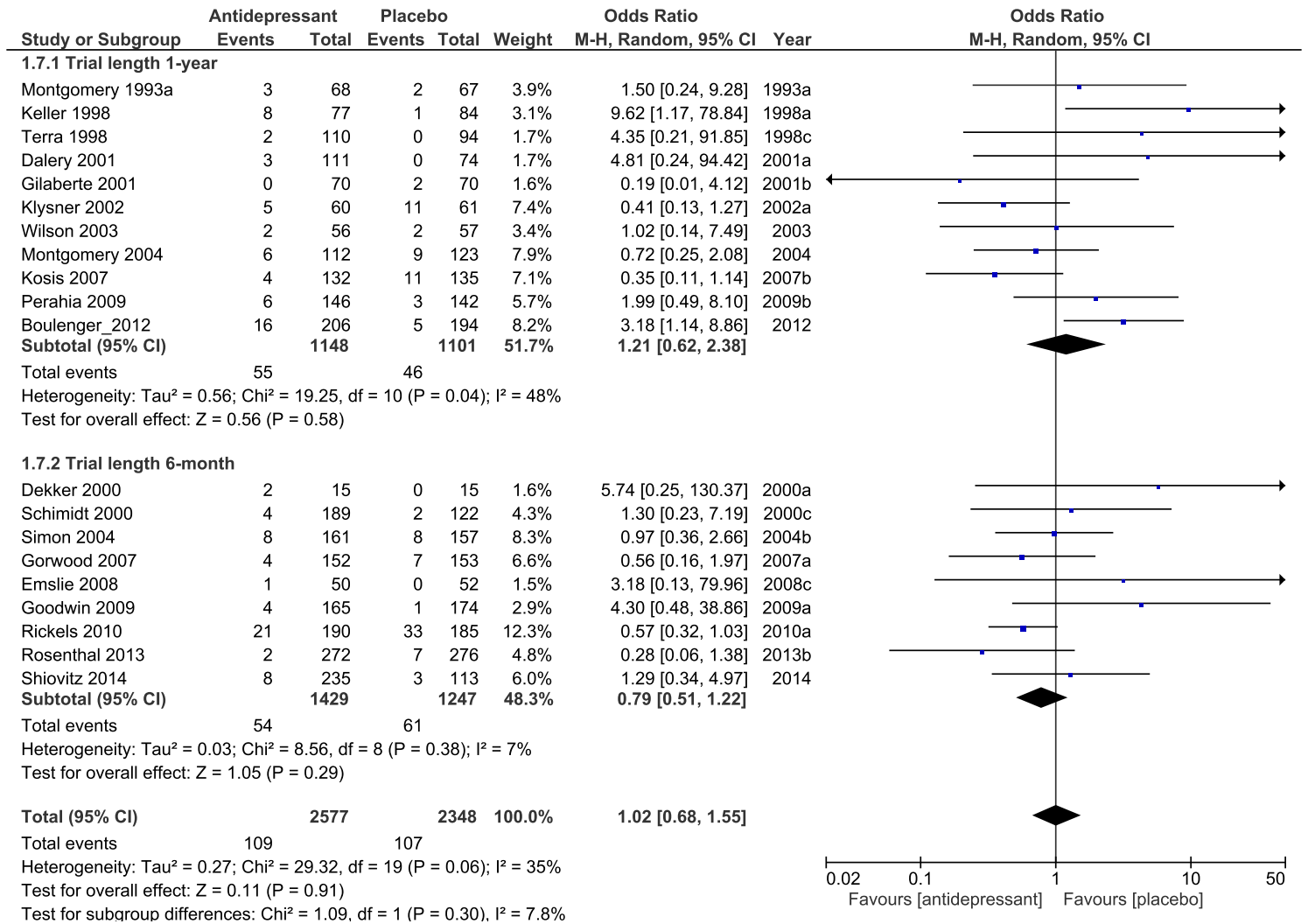

Supplement: Supplementary file 8 — Supplemental Figure 5 [file 41380_2020_843_MOESM8_ESM.pdf]

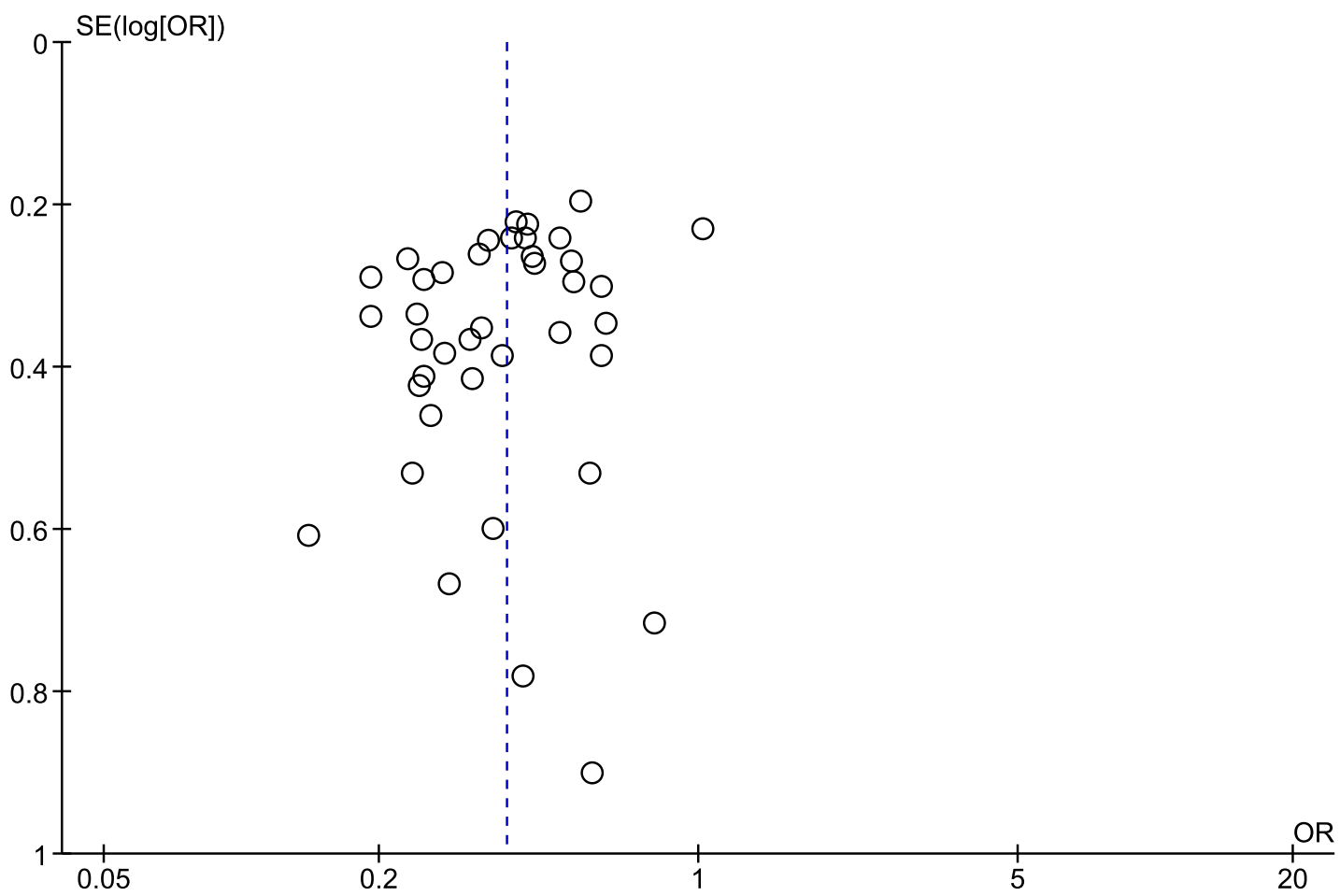

Supplement: Supplementary file 9 — Supplemental Figure 6a [file 41380_2020_843_MOESM9_ESM.pdf]

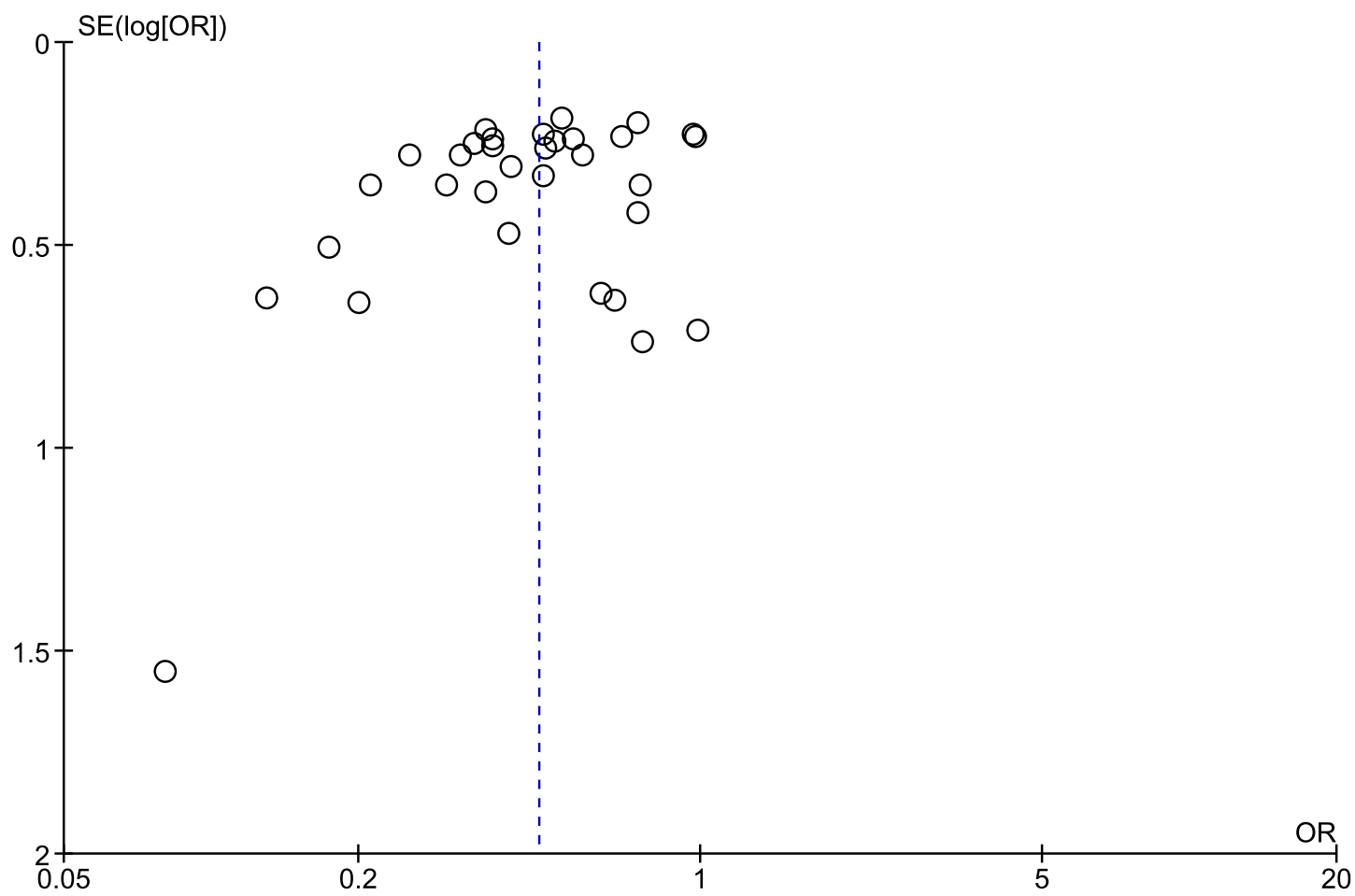

Supplement: Supplementary file 10 — Supplemental Figure 6b [file 41380_2020_843_MOESM10_ESM.pdf]
